# Evaluation of Commercial Circulating Tumor DNA Test in Metastatic Prostate Cancer

**DOI:** 10.1200/PO.19.00014

**Published:** 2019-06-12

**Authors:** Sinja Taavitsainen, Matti Annala, Elisa Ledet, Kevin Beja, Patrick J. Miller, Marcus Moses, Matti Nykter, Kim N. Chi, Oliver Sartor, Alexander W. Wyatt

**Affiliations:** ^1^ University of British Columbia, Vancouver, British Columbia, Canada; ^2^ Tampere University, Tampere, Finland; ^3^ Tulane University, New Orleans, LA; ^4^ British Columbia Cancer Agency, Vancouver, British Columbia, Canada

## Abstract

**PURPOSE:**

Circulating tumor DNA (ctDNA) sequencing provides a minimally invasive method for tumor molecular stratification. Commercial ctDNA sequencing is increasingly used in the clinic, but its accuracy in metastatic prostate cancer is untested. We compared the commercial Guardant360 ctDNA test against an academic sequencing approach for profiling metastatic prostate cancer.

**PATIENTS AND METHODS:**

Plasma cell-free DNA was collected between September 2016 and April 2018 from 24 patients with clinically progressive metastatic prostate cancer representing a range of clinical scenarios. Each sample was analyzed using Guardant360 and a research panel encompassing 73 prostate cancer genes. Concordance of somatic mutation and copy number calls was evaluated between the two approaches.

**RESULTS:**

Targeted sequencing independently confirmed 94% of somatic mutations identified by Guardant360 at an allele fraction greater than 1%. *AR* amplifications and mutations were detected with high concordance in 14 patients, with only three discordant subclonal mutations at an allele fraction lower than 0.5%. Many somatic mutations identified by Guardant360 at an allele fraction lower than 1% seemed to represent subclonal passenger events or non–prostate-derived clones. Most of the non-*AR* gene amplifications reported by Guardant360 represented single copy gains. The research approach detected several clinically relevant DNA repair gene alterations not reported by Guardant360, including four germline truncating *BRCA2*/*ATM* mutations, two somatic *ATM* stop gain mutations, one *BRCA2* biallelic deletion, 11 *BRCA2* stop gain reversal mutations in a patient treated with olaparib, and a hypermutator phenotype in a patient sample with 42 mutations per megabase.

**CONCLUSION:**

Guardant360 accurately identifies somatic ctDNA mutations in patients with metastatic prostate cancer, but low allele frequency mutations should be interpreted with caution. Test utility in metastatic prostate cancer is currently limited by the lack of reporting on actionable deletions, rearrangements, and germline mutations.

## INTRODUCTION

Molecular stratification is poised to guide treatment of metastatic prostate cancer (mPC), but tissue biopsies are difficult to acquire, because many patients lack soft tissue metastases. Fortunately, circulating tumor DNA (ctDNA) can be detected in peripheral blood cell–free DNA (cfDNA) from most patients with progressive disease.^1-4^ Numerous research, industry, and commercial cfDNA sequencing assays have arisen, each aiming to provide a practical alternative to metastatic tissue biopsy for tumor molecular stratification. Unlike many other solid cancers, where ctDNA assays can rely on detection of recurrent clinically relevant mutations,^5^ mPC is characterized by a low mutation rate and high prevalence of large structural rearrangements, including deletions and translocations.^6^ Furthermore, up to 30% of patients with mPC harbor deleterious germline and/or somatic defects in DNA damage repair genes, such as *BRCA2.*^7^

**CONTEXT**

**Key Objective**
To evaluate a commercial circulating tumor (ctDNA) test by comparing it against a prostate cancer–specific research panel in matched same-day plasma samples from men with metastatic prostate cancer.
**Knowledge Generated**
High concordance between the approaches was observed for *AR* gene copy number calls and somatic mutations at an allele fraction greater than 1%. Most low allele fraction mutations reported by the commercial test seemed to represent subclonal passenger mutations or non–prostate-derived mutations. The commercial test did not report several clinically actionable DNA repair defects, including *BRCA2* somatic homozygous deletions, structural rearrangements, and germline truncating mutations.
**Relevance**
Our results suggest that clinicians should use caution when interpreting commercial ctDNA test results, particularly in the context of low allele fraction mutations. Test utility in prostate cancer is limited by the lack of reporting on germline mutations, somatic deletions, and structural variants.

The Clinical Laboratory Improvement Amendments–certified Guardant360 ctDNA test (Guardant Health, Redwood City, CA) identifies single-nucleotide variants (SNVs) in 73 genes, insertions/deletions (indels) in 23 genes, copy number amplifications (CNAs) in 18 genes, and gene fusions in six genes. It is widely used for characterization of mPC and other solid cancers.^8^ Recently, low congruency was reported between mPC samples submitted for parallel testing with Guardant360 and another commercial ctDNA assay, PlasmaSELECT (Personal Genome Diagnostics, Baltimore, MD).^9^ A separate study examining breast and other solid cancers subjected matched patient specimens to Guardant360 ctDNA testing and FoundationOne (Foundation Medicine, Cambridge, MA) tumor tissue testing and also found high discordance, particularly for mutations reported at allele frequencies lower than 1%.^10^ Together, these results raise significant concerns about the accuracy of ctDNA testing in precision oncology and are consistent with recent consensus statements on insufficient evidence of clinical validity for ctDNA assays in advanced cancer^11^ and lack of support for routine ctDNA testing in mPC.^12^

Given the increasing use of commercial ctDNA testing, we sought to assess the strengths and limitations of Guardant360 in mPC. We previously demonstrated high concordance for somatic alterations detected in ctDNA and matched metastatic tissue using a PC-specific targeted sequencing approach applied in a research setting.^13^ Therefore, in the study reported here, we performed a blinded analysis of 24 cfDNA samples subjected to Guardant360 testing and our academic cfDNA sequencing approach.

## PATIENTS AND METHODS

### Blood Collection and Guardant360 Testing

Peripheral venous blood was collected between September 2016 and April 2018 from 24 patients with progressive castration-resistant mPC (Data Supplement). Blood was collected in two 10-cc Streck tubes following Guardant Health standard collection protocol and shipped for testing at Guardant Health. Guardant360 uses digital sequencing to detect SNVs, indels, CNAs, and fusions in select exons and genes from cfDNA. All genomic alterations reported by Guardant360 in each of the 24 patients with mPC were used in this study. Raw Guardant360 sequencing data were not available for bioinformatic analysis, and there was no research agreement with Guardant Health.

Matched same-day samples from the 24 patients with mPC sent for Guardant360 testing were subjected to targeted cfDNA and WBC sequencing using a previously published academic approach (the Vancouver panel).^2,13^ Targeted cfDNA and WBC sequencing and data analysis were performed and finalized before examination of Guardant360 reports. Approval for this study was granted by the Tulane University Institutional Review Board (certificate M0600) and the University of British Columbia Clinical Research Ethics Board (certificate H18-00944). The study was conducted in accordance with the Declaration of Helsinki, and written informed consent was obtained from all participants before enrollment.

### Sequence Alignment and Quality Control

Paired-end reads (from the Vancouver panel) were aligned against an hg38 reference genome using Bowtie (version 2.3.0).^14^ For WBC samples, duplicate reads were marked using samblaster (version 0.1.24).^15^ For cfDNA samples that carried 4-bp unique molecular identifiers, reads were marked as duplicates if they were aligned to the same position and their 4-bp unique molecular identifiers had at least three identical bases. Adapters in read 3′ ends were trimmed in paired mode using cutadapt (version 1.11).^16^ Low-quality read tails (smoothed baseq < 30) were trimmed using an in-house algorithm. Per-base read coverages in target regions were counted using bedtools (version 2.25.0).^17^ cfDNA/WBC sample pairings were verified based on SNP genotypes.

### Analysis of Somatic Mutations

Somatic mutations were called in cfDNA samples by searching for variants with at least eight supporting reads and a mutant allele fraction (AF) of at least 0.5%. The AF was required to be at least 20 times higher than the average AF across all WBC samples and at least three times higher than the AF in the paired WBC sample. The paired WBC sample had to have at least 20 reads covering the position. For base substitutions, the average mapping quality of mutation supporting reads was required to be at least 10, and the average distance of the mutant allele from the nearest read end was required to be at least 15 bases. Our mutation analysis pipeline was previously validated in matched tissue-cfDNA cohorts^13,18^ and in dilution series experiments.^2^ Protein-level consequences of variants were predicted using ANNOVAR.^19^ ctDNA fractions were estimated based on somatic mutation AFs as previously described.^2^

Mutations were considered subclonal if their AF was less than 0.25 times the ctDNA fraction (for autosomes) or less than 0.5 times the ctDNA fraction (for chromosome X). This conservative threshold was determined by halving the expected AF for truncal mutations.

**FIG 1.**
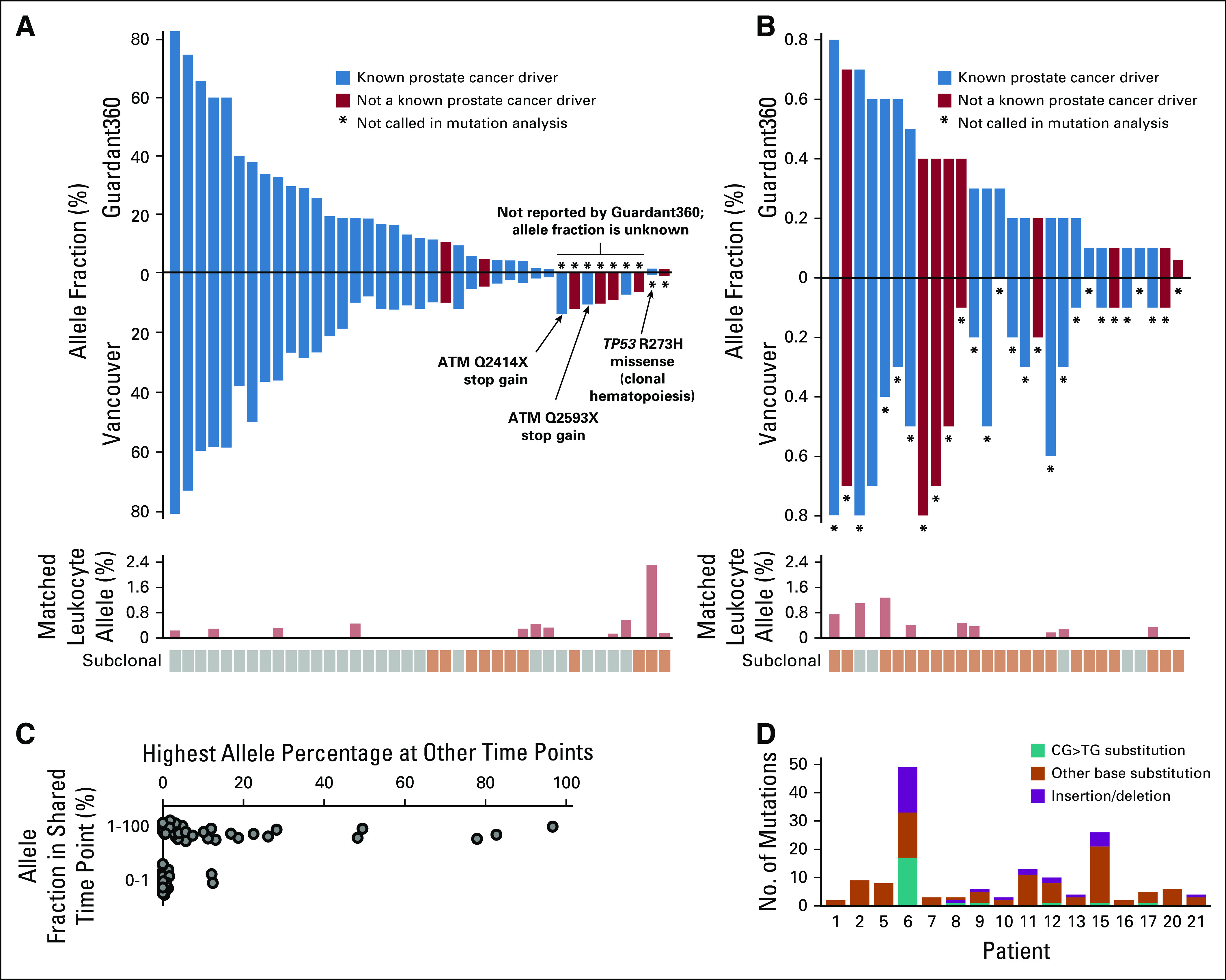
Concordance of somatic circulating tumor DNA mutation calls between Guardant360 and the Vancouver panel. Allele fractions of somatic mutations based on the two assays: mutations with allele fraction of (A) 1% or greater and (B) lower than 1%. Known prostate cancer driver mutations are shown in blue; other mutations in red. Bar plot below shows allele fraction in matched WBC samples. Mutations were labeled as subclonal if their allele fraction was less than half the allele fraction expected for truncal mutations (described in Patients and Methods). It is plausible that some mutations labeled as subclonal had a nonprostate origin. (C) Plot showing all mutations (dots) identified by Guardant360 in the cell-free DNA (cfDNA) time points that were also analyzed with the Vancouver panel, grouped by allele fraction. Position along *x*-axis indicates the highest allele fraction that those mutations reached in other time points analyzed by Guardant360. (D) Bar plot showing the total number of somatic mutations called by the Vancouver panel in 16 cfDNA samples with detectable mutations. Patient 6 displays a hypermutation signature enriched for somatic CG>TG transitions and insertions/deletions, consistent with an underlying mismatch repair defect.

Two *TP53* missense mutations in the cohort had an AF greater than 1% in cfDNA and a similar AF in the matched WBC sample. Because these mutations likely originated from an expanded mutant WBC clone (clonal hematopoiesis), we rejected them as somatic PC mutations. One of these mutations (a *TP53* p.R273H missense mutation in patient 5) was reported by Guardant360 and is included in Figures 1A and 1B and the Data Supplement.

### Analysis of Deleterious Germline Variants

Germline variants were called in WBC samples by searching for variants with an alternate AF of at least 15% and at least eight supporting reads. Germline variants with a population allele frequency of 0.5% or higher in the KAVIAR (Known Variants) or ExAC (Exome Aggregation Consortium) database were discarded. Protein-level consequences of variants were predicted using ANNOVAR.^19^

### Copy number Calling

Reads were counted in all target regions using bedtools (version 2.25.0).^17^ A control cfDNA sample from a healthy volunteer was used as a reference for GC correction. GC fraction was calculated for all target regions, and a scatter plot was created for each sample showing target regions as dots, with coverage log ratio relative to golden reference on the *y*-axis and GC fraction on the *x*-axis. Loess regression was applied to the scatter plot to normalize GC bias across all samples. After GC correction, a median reference profile representing noncancerous cfDNA was constructed based on 138 cfDNA samples with no detectable somatic mutations or amplifications. The final coverage log ratio for each gene was calculated as the median coverage log ratio of all target regions inside the gene. Our copy number analysis pipeline was previously validated in a matched tissue-cfDNA cohort.^13^

A deletion was called for a gene when the coverage log ratio was −0.3 or lower. A gain was called for a gene when the coverage log ratio was 0.3 or higher. These conservative thresholds were determined empirically by studying a plot of coverage log ratios and heterozygous SNP AFs in samples with and without detectable ctDNA.

### Categorization of Putative PC Driver Alterations

The following somatic mutations detected in the cohort were considered putative PC drivers: *TP53* missense and truncating mutations; *AR* missense mutations in amino acids 702, 742, 875, 878, and 893; *AKT1* p.E17K missense mutations; *APC* truncating mutations; *BRAF* missense mutations in amino acids 600 and 601; *PTEN* missense mutations in amino acid 35; *PIK3CA* missense mutations in amino acids 378, 391, and 1047; *ATM* truncating mutations; *CTNNB1* missense mutations in amino acids 32 to 45; and *RB1* truncating mutations and missense mutations in amino acid 661. Putative driver mutations were determined from review of recently published large castration-resistant mPC sequencing studies.^6,20,21^

### Analysis of Serial Blood Collections

In addition to the matched 24 same-day samples subjected to Guardant360 testing and Vancouver panel sequencing, Guardant360 reports were available for an additional 86 blood collections from the same patients collected between August 2015 and July 2018. Note that throughout the course of the study time period, three versions of the Guardant360 assay (68-, 70-, and 73-gene panels) were used, with expanding coverage of genes and alterations. All somatic mutations identified as discordant in this study were confirmed to have been tested with the Guardant360 73-gene panel.

## RESULTS

### Concordance of Somatic Mutation Calls

Our objective was to compare the Guardant360 test against a PC-specific ctDNA research assay (Vancouver panel) that identifies SNVs, indels, amplifications, deletions, and rearrangements in the exonic regions of 73 mPC-relevant genes (Data Supplement).^2,13,22^ Between September 2016 and April 2018, we sent plasma from 24 patients with clinically progressive mPC for Guardant360 testing and performed targeted sequencing on matched same-day samples using the Vancouver panel (median depth, 1,435×; data deposited to European Genome-Phenome Archive under accession EGAS00001003352; Data Supplement). Four patients had no detectable ctDNA according to both assays. Across the remaining 20 patients, ctDNA fractions estimated by the Vancouver panel ranged between undetectable (< 2%) and 80% (median, 23%; Data Supplement).

Focusing on 26 genes covered by both approaches, 30 (94%) of 32 somatic mutations identified by Guardant360 with an AF greater than 1% were independently confirmed by the Vancouver panel (Figs 1A and 1B; Data Supplement). Of the two discordant mutations, one was also found in matched WBCs, and one had a low AF (0.8%) in our analysis. Guardant360 also reported 28 somatic mutations with an AF lower than 1%; previous reports have expressed concern regarding AFs below this threshold.^10^ We found supporting reads for 24 (86%) of these mutations in cfDNA, but nine of the mutations were found at a similar AF in matched WBCs (which are not analyzed by Guardant360; Figs 1A and 1B). Across all Guardant360 panel genes, 81% of mutations with an AF greater than 1% had a putative driver role in PC, compared with 41% of mutations with an AF lower than 1% (Fisher’s exact test *P* < .001). Furthermore, 82% of mutations with an AF lower than 1% were found to be subclonal (Figs 1A and 1B; criteria described in Patients and Methods). To explore further, we examined serial Guardant360 test results from the same patients and found that 96% of mutations detected at an AF lower than 1% did not reach an AF greater than 2% at any time point (Fig 1C) and did not track with overall ctDNA fraction (Data Supplement). These data suggest that many low AF mutations identified by Guardant360 represent subclonal passenger events or rare somatic clones of nonprostate origin.

Guardant360 did not report seven of 39 somatic mutations identified by the Vancouver panel at allele fractions between 6% and 14% (median, 10%; Data Supplement). The Vancouver panel also detected a high somatic mutation burden (42 mutations per Mb) in patient 6 (Fig 1D), accompanied by *MSH2* and *MSH6* monoallelic losses. Guardant360 does not report silent mutations or total mutation burden. This patient had a complete remission (9 months at last follow-up) after pembrolizumab treatment.

### Sensitivity Toward Somatic Changes in *AR*

**FIG 2.**
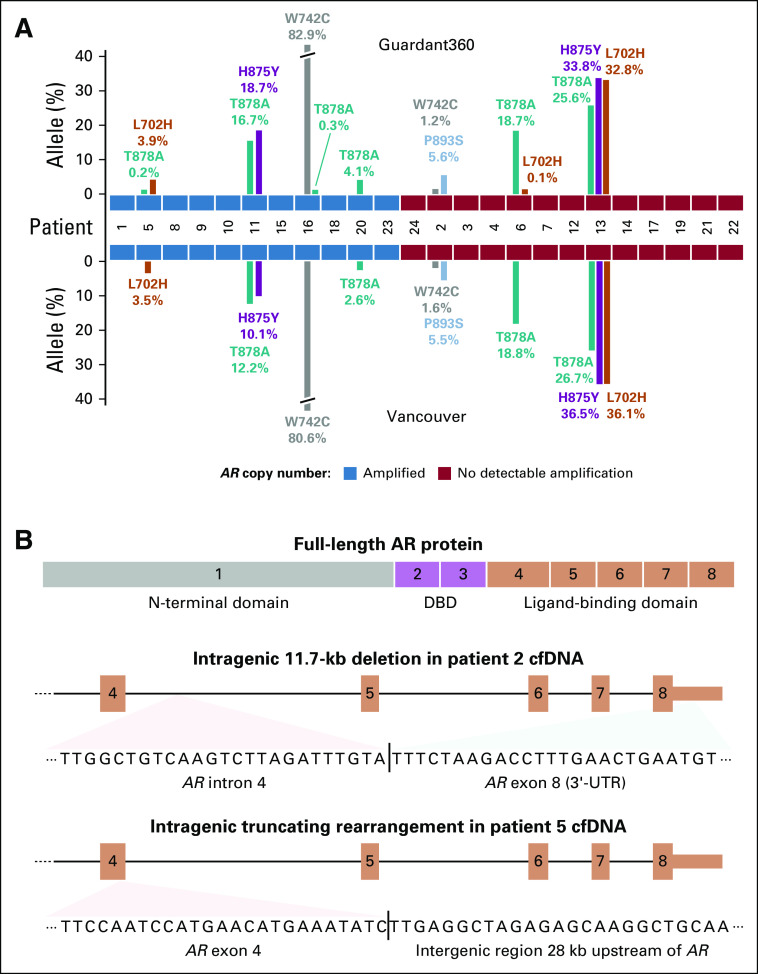
Genomic changes identified in *AR* by Guardant360 and the Vancouver panel. (A) Bar plot showing allele fractions of somatic *AR* hotspot mutations detected by the two assays. *AR* amplification status is indicated by blue and red tiles in the middle. (B) Structure of two androgen receptor (AR) ligand-binding domain truncating rearrangements identified by the Vancouver panel. The somatic junction sequences detected in cell-free DNA (cfDNA) are shown below. At the top, a diagram of full-length AR protein highlights that exons 4 to 8 code for the ligand-binding domain. DBD, DNA binding domain.

The *AR* gene is altered in most treatment-resistant mPCs, and *AR* status is associated with therapy response.^1,2,6,23^ In this cohort, *AR* amplifications were identified in 11 of 24 patients, with perfect concordance between assays. Somatic hotspot mutations in the androgen receptor (AR) ligand-binding domain were also highly concordant, except for three *AR* mutations detected by Guardant at an AF lower than 0.5% (Fig 2A). These mutations had supporting reads in our assay and were likely subclonal based on the presence of other mutations at higher AFs (Data Supplement). Guardant360 does not report structural variants within the *AR* gene. We identified two patients with AR ligand-binding domain rearrangements predicted to yield a constitutively active AR protein (Fig 2B).^24^

### Identification of Actionable DNA Repair Gene Alterations

**FIG 3.**
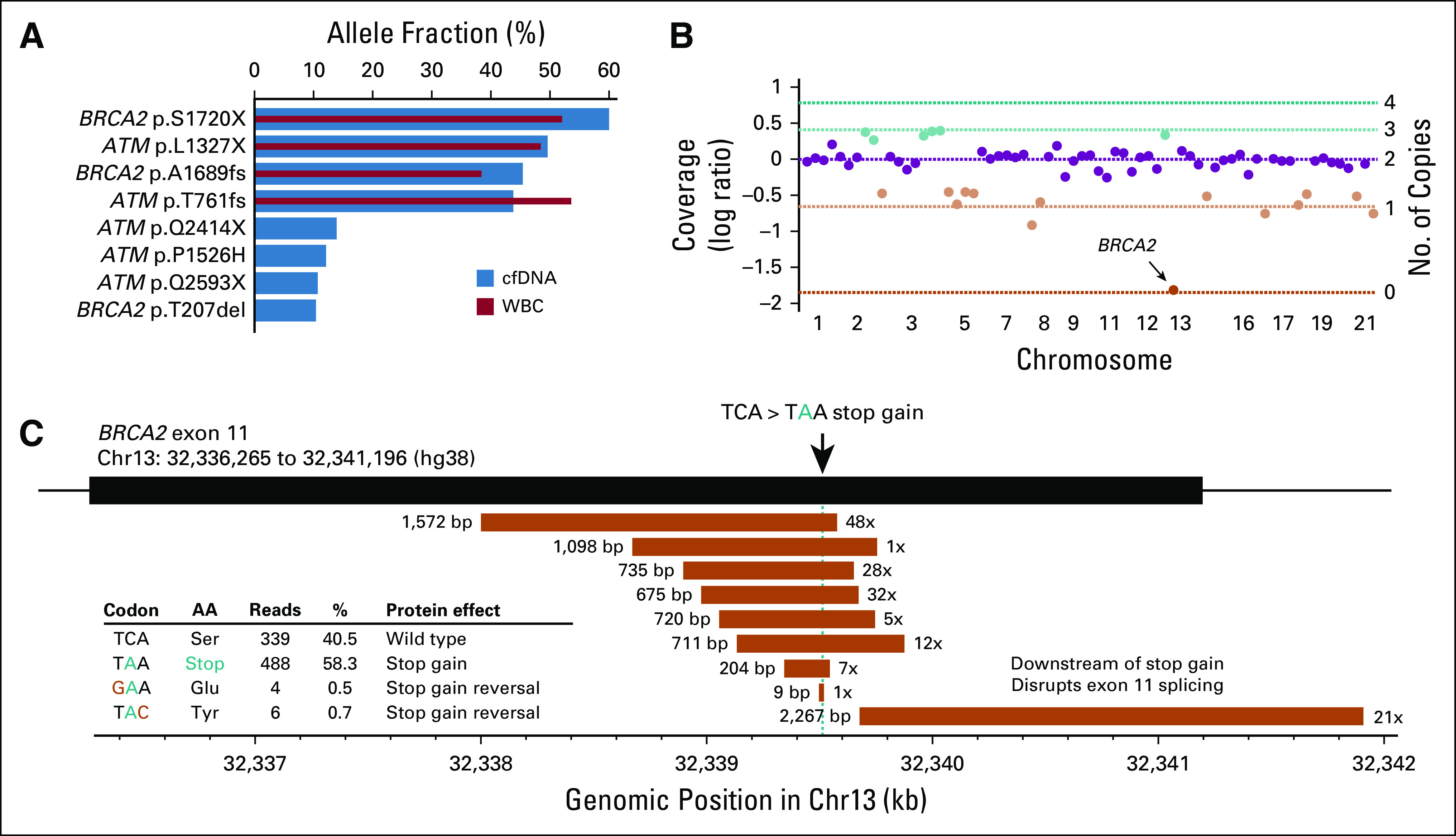
Homologous recombination repair defects identified by the Vancouver panel. (A) Bar plot showing allele fractions of four germline and four somatic *BRCA2* or *ATM* truncating mutations identified by the Vancouver panel but not reported by Guardant360. Blue and red bars show mutant allele fraction in the cell-free DNA (cfDNA) sample and matched WBC sample, respectively. (B) Scatter plot showing sequencing coverage log ratios (Vancouver panel) for all 69 autosomal genes in cfDNA of patient 12. Horizontal lines indicate expected log ratios for different copy numbers, given his estimated ctDNA fraction of 72%. Green and orange colors indicate copy number gains and losses, respectively. *BRCA2* biallelic loss in chromosome 13 (chr13) is indicated with an arrow. (C) Eleven *BRCA2* stop gain reversing somatic alterations detected with the Vancouver panel in cfDNA from patient 17 after treatment with poly (ADP-ribose) polymerase inhibitor olaparib. Deletions are shown as orange rectangles, with length (in base pairs [bp]) shown on the left and number of supporting reads on the right. All deletions overlapping the stop gain mutation are multiple of 3 bp in length and therefore remove the stop gain mutation while avoiding frameshift. Stop gain–reversing base substitutions are shown in the embedded table.

Deleterious alterations in homologous recombination repair genes are associated with response to poly (ADP-ribose) polymerase (PARP) inhibitors and platinum-based chemotherapy.^7,25^ The Vancouver panel identified six patients with homologous recombination repair defects: four patients with a germline *BRCA2* or *ATM* truncating mutation (independently verified by commercial germline testing), two patients with somatic *ATM* stop gain mutations, and one patient with a somatic *BRCA2* biallelic deletion (Figs 3A and 3B). Guardant360 does not report germline mutations or somatic copy number losses and surprisingly did not report the two somatic *ATM* stop gain mutations. Notably, patient 17 was sampled after olaparib resistance in March 2018, and his cfDNA revealed 11 cancer cell populations with somatic deletions or mutations reversing the germline *BRCA2* stop gain (Fig 3C).^26^ These alterations were not reported by Guardant360, likely because it only reports short indels.

### Concordance of Gene Amplification Calls

**FIG 4.**
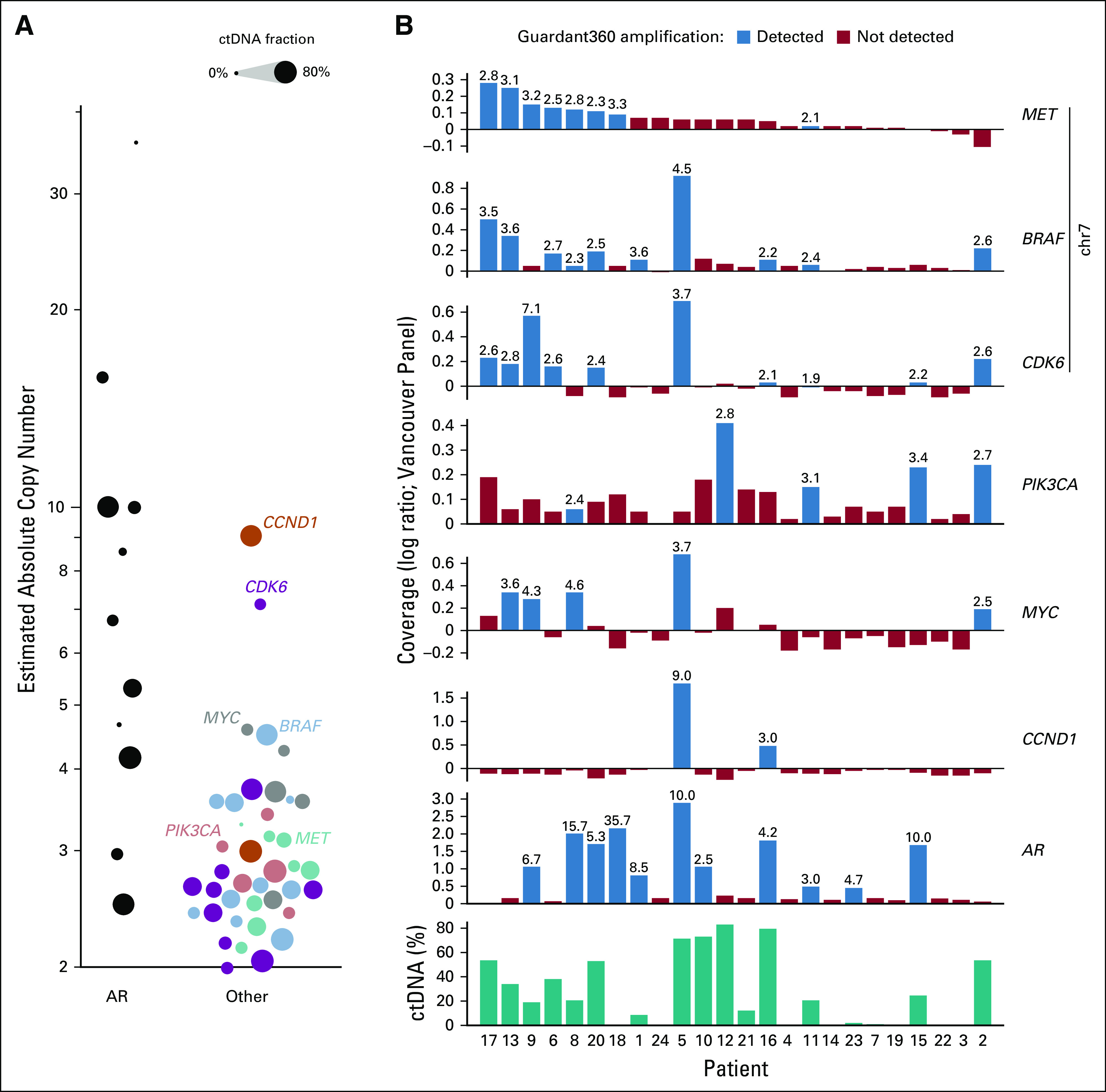
Concordance of gene amplification calls between Guardant360 and the Vancouver panel. (A) Absolute gene copy number (estimated with the Vancouver panel) of *AR* compared with six other genes. Copy numbers were estimated based on sequencing coverage log ratio and circulating tumor (ctDNA) fraction (described in Patients and Methods) and represent the average gene copy numbers in ctDNA-shedding cancer cells. Circle size represents the estimated ctDNA fraction of a sample. (B) Sequencing coverage log ratios of genes *MET*, *BRAF*, *CDK6*, *PIK3CA*, *MYC*, *CCND1*, and *AR*, quantified with the Vancouver panel in all 24 cfDNA samples. Bars highlighted in blue indicate that the gene was reported as amplified by Guardant360 in that sample. Numbers above bars indicate the average gene copy numbers in ctDNA-shedding cancer cells that would produce the observed coverage log ratio in the Vancouver panel data, correcting for presence of normal cfDNA (Patients and Methods). Chr, chromosome.

Amplification of chromosome 7 genes *MET*, *BRAF*, and *CDK6* is rare in mPC, although low-level gain of chromosome 7 is common.^6,21^ *MET*, *BRAF*, and/or *CDK6* was reported amplified by Guardant360 in 13 patients. Our analysis supported the presence of chromosome 7 single copy gain in many of these patients (Fig 4A). Guardant360 additionally reported 12 amplifications in *PIK3CA*, *CCND1*, and *MYC*, nine of which showed evidence consistent with single copy gain. Only 11 of 40 reported non-*AR* amplifications displayed evidence for more than a single copy gain (Fig 4B). Guardant360 also reported 11 amplifications in genes *EGFR*, *PDGFRA*, *KIT*, and *FGFR1*, which are rarely altered in tissue-based mPC studies^6^ and are not assessed by the Vancouver panel.

Guardant360 does not search for alterations in mPC genes *SPOP*, *CDK12*, *PIK3R1*, *FOXA1*, *MSH2*, *MSH6*, *TMPRSS2*, and *ERG* and does not report large somatic deletions or complex structural variants in any genes. In total, we identified potentially clinically relevant alterations that were not reported by Guardant360 in 12 of 24 patients, including a *TP53* inactivating rearrangement, a *CDKN1B* biallelic deletion, intragenic *AR* rearrangements in two patients, three *SPOP* mutations, and DNA repair defects in seven patients (Data Supplement).

## DISCUSSION

The Guardant360 commercial ctDNA assay has improved access to genomic testing across academic and nonacademic practitioners, especially in settings where tissue biopsy is not feasible. As with any narrow pan-cancer assay, there are compromises for individual cancer types.

Overall, we found excellent concordance between Guardant360 and the Vancouver PC panel for high AF somatic mutations. Guardant360 also exhibited high sensitivity for low AF (< 1%) mutations, although our results suggest these mutations must be interpreted with caution. First, a vast majority of these mutations were subclonal and therefore potentially poor biomarkers for therapies aiming at broad antitumor effect, although they may still be of relevance for detecting emerging resistant clones. Second, low AF mutations were more likely to have features consistent with passenger status and therefore less relevance to therapy resistance or response. Third, approximately one third of low AF mutations had a similar level of read support in the WBC fraction, suggestive of a clonal hematopoietic origin. It is well established that elderly populations such as those affected by PC have a high prevalence of clonal hematopoiesis.^27,28^ Indeed, a recent study of cfDNA from 217 patients with mPC suggested that 15% would have somatic alterations falsely attributed to PC-derived ctDNA if the WBC fraction were not analyzed.^3^ Because Guardant360 only analyzes cfDNA, the test cannot reject somatic cfDNA mutations that are simultaneously detected in the blood lineage. Consistent with these three points, a vast majority of low AF mutations did not track with overall ctDNA fraction and never rose to a significant clonal fraction in longitudinal sampling. Finally, earlier studies comparing commercial ctDNA assays found large discordances in reported low AF somatic mutations.^10^ Together, these results suggest that Guardant360 should consider differential reporting of low AF mutations, especially for mPC.

Somatic changes to the *AR* gene, including mutations, amplifications, and rearrangements resulting in constitutive activation, are primary drivers of resistance to systemic therapies targeting the androgen axis in PC^24^ and are under development as predictive cfDNA biomarkers.^2,29,30^ We found near-perfect concordance between Guardant360 and the Vancouver panel for *AR* amplifications and hotspot mutations, with the only limitation being that Guardant360 does not currently report intragenic *AR* rearrangements. The Vancouver panel identified two patients with a potentially ligand-independent AR in this cohort.

Guardant360 does not report germline alterations, somatic copy number deletions, or large structural variants in the DNA damage repair genes included in its panel. In mPC, these classes of variants are common, especially across *BRCA2*, and can have both prognostic and predictive implications.^31^ Of particular relevance, biallelic *BRCA2* defects can sensitize to PARP inhibitors, whereas deletions overlapping the site of a pre-existing mutation (ie, so-called reversion mutations) can drive PARP inhibitor resistance.^26,32^ One quarter of the patients profiled here had biologically and clinically relevant DNA damage repair gene alterations identified via sequencing with the Vancouver panel. Because nongenomic specialists may not be aware of the limits of commercial assays, we caution that negative results from Guardant360 should be interpreted critically; absence of reported somatic mutation in a gene does not preclude other types of alterations (eg, somatic biallelic *BRCA2* deletion, rearrangement, or germline truncating mutation).

In conclusion, we show that Guardant360 and targeted ctDNA sequencing with a research assay (ie, the Vancouver panel) are highly concordant for high AF mutations in mPC. However, the Guardant360 test has potential limitations in its reporting of indels, rearrangements, and germline variants. Such variants are likely informative for guiding patient treatment. In the future, optimal ctDNA commercial assays for mPC should identify and report on all types of somatic alterations, including deletions and rearrangements; include mPC-specific genes, such as *FOXA1*, *SPOP*, and *ERG*; and identify deleterious germline alterations. Limitations of this study include the absence of matched tissue to adjudicate discordances and the lack of a standardized cohort to draw clinical outcome correlates.
